# Editorial: Cognition During Sleep: Hyperassociativity, Associativity and New Connections

**DOI:** 10.3389/fpsyg.2020.641547

**Published:** 2021-01-22

**Authors:** Caroline L. Horton, Sue Llewellyn

**Affiliations:** ^1^DrEAMSLab, Bishop Grosseteste University, Lincoln, United Kingdom; ^2^Faculty of Humanities, The University of Manchester, Manchester, United Kingdom

**Keywords:** sleep, cognition, consciousness, hyperassociativity, memory, consolidation

The purpose of this collection was to collate evidence and emerging ideas from the neurosciences, cognitive sciences, and consciousness studies to understand the nature of cognition in sleep. In particular, within the literature on dreaming, cognitive processes of “hyperassociativity,” or “loose connections,” have been suggested to be a key feature of Rapid Eye Movement (REM) sleep and dreaming. Hyperassociativity has been ill-defined, though it seems to comprise diffuse, surprising or weakly-linked memories or memory fragments, which are activated either sequentially or in parallel during a dream. Recent scholars have proposed that hyperassociativity may create the environment in which novel insights, new solutions and creativity can flourish and may therefore underpin some of the cognitive benefits of REM sleep. REM dreaming, in particular, may have evolved to spot non-obvious, remote associations which, coalesce to visualize probabilistic patterns in past events.

The papers within this Research Topic explored and considered cognition during sleep from several angles.

Nordin and Bjälkebring(a) sparked a debate within the issue concerning the nature of cognition within REM sleep, as contextualized as the supernatural agent (a facet of self) in dreams, with Sears responding with a critical dismissal of the underpinning theory, and the original authors [Nordin and Bjälkebring(b)] responding again. The debate concerned the nature of counterintuitiveness in dream imagery, what such cognitive structures could represent and how it could be measured. Whilst the original authors [Nordin and Bjälkebring(a)] made use of a coding scheme devised by Barrett (2008) for the first time, Sears raised some methodological and conceptual criticisms of this. The debate highlighted the need for further clarity surrounding measures of dreaming cognition, as well as the range of approaches to studying it within dream science.

Barcaro et al. illustrated the complexity of cognition underlying consciousness in terms of dream formation and possible purpose, with three dreams. They considered the parallel processes involved with hyperassociativity, i.e., the simultaneous activation and combination of several memory sources, along with the activation—or perhaps even suspension of—present concerns (as such concerns seem to motivate the dreamer toward reducing the negative emotionality associated with those experiences). However, such complex parallel processes lead to an apparently serial presentation of dream images within the dream scenario. The authors must be congratulated for synthesizing theories of consciousness and cognition as a means of accounting for dream formation. We suggest that hyperassociativity allows for the presentation of these numerous memory sources in dreams, or sleeping cognition, potentially reconciling these models.

Hołda et al. explored the effect of a 90-min nap, relative to comparable wakefulness, of problem solving using a real-world task featuring the presentation of a crime story via an interactive computer task. Sleep architecture revealed that all participants experienced REM as well as all non-REM stages. Contrary to expectations, no effect of sleep on problem solving was found. Neither quality nor creativity of the solutions generated by the participants was higher in the nap group than in the waking group, though naps only lasted around an hour on average.

Similarly Solomonova et al. did not identify global benefits of sleep on cognitive outcomes (in this case a procedural memory consolidation task), but rather, a more nuanced one. They compared meditation practitioners with non-meditating controls on both the memory outcomes and sleep architecture. Whilst architecture was comparable overall, task performance correlated with spindle activity in the meditation group, but with REM in the control group. The authors speculate that meditation may alter sleep architecture in response to learning and memory, which indicates possible learned mechanisms for reorganizing neural architecture. The possibly more dynamic, rather than fixed, nature of cognition during sleep is therefore suggested.

Fogel et al. also investigated relationships between sleep and learning by exploring dream content in response to recent prior learning, as mediated by inter-individual reasoning (for early dreams) and verbal abilities (for later dreams). Dreams reported from early in the night reflected the extent of learning within the reasoning task. In that way the findings demonstrated possible links between dream production and manifestation, and specific cognitive styles during wake.

Vallat et al. explored group trait differences in dream recall. They demonstrated that medial prefrontal cortex white matter density was greater in high dream recallers compared to low dream recallers. Whether the anatomical differences result from learning or are congenital, we cannot say, however accumulated evidence indicates the importance of the medial prefrontal cortex in dream recall—or perhaps dream production. The more evidence we can accumulate concerning the neural substrates underlying dreaming, the better we may be able to make inferences about the large-scale function of dreaming, particularly in relation to memory- or emotion-processing.

Continuing this exploration of traits, Blagrove et al. considered both state and trait empathy as related to dream sharing. Trait empathy was found to be significantly associated with the frequency of listening to the dreams of others, frequency of telling one's own dreams to others, and attitude toward dreams. In a second study, dream sharing increased state empathy, leading the authors to suggest that these relationships between dreaming and sharing of experiences may afford us the opportunity to use dreams to facilitate social bonding. In this way, the sleep mentation may be functional. It may also be open to change or distortion, reiterating Solomonova et al.'s sentiments above that sleeping cognition may not be fixed.

Kahn explored continuity in terms of the self and noted that the cognitive reactions to activity during sleep, in the context of dream activity, was somewhat discontinuous with waking life but continuous with the dream activity, such that dream content was bizarre by waking standards, but it was largely accepted during the dream. Kahn gives examples of dream content whereby there are changes in situations, or hyperassociativity of places and times, but that these are accepted by the dream-self. Thus, hyperassociative cognition during sleep seems to be a “normal” part of activity at that time. This acceptance may also hint at the function (or functions) of hyperassociativity.”

Rozen and Soffer-Dudek investigated the physiological and psychological correlates of a specific yet common dream event: that of teeth falling out, in an attempt to explore the continuity hypothesis. Teeth dreams were associated with specific measures of current dental distress and irritation, whereas other (non-teeth dreams) were not associated with such dental distress. Whilst this does not support the continuity hypothesis in terms of memories, or recent events, being reactivated during sleep and consequently manifesting in dreams, it does support the continuity of perceptions and concerns, whilst going against the more symbolic notions that teeth dreams may be a representation of something non-physiological. This clear and helpful illustration of a specific dream theme has helped to elucidate some of the more universal cognitive mechanisms underlying dream production.

Taken together, this collection has explored the cognitive correlates of sleep and dreaming, articulating the apparently more universal nature of hyperassociativity for the first time. We propose that identifying cognitive features of sleep over the course of the night may provide insights as to sleep's function, whilst recognizing the challenges of studying dreams more subjectively. We suggest that hyperassociativity be studied further, with particular reference to the nature of the links between memory sources being identified over the course of the night.

## Author Contributions

Both authors contributed to the development of the ideas underlying the Research Topic concerning hyperassociativity and both engaged with the Topic as editors. CH drafted the editorial, on the basis of the manuscripts both authors had reviewed, discussed, and revised it prior to submission.

## Conflict of Interest

The authors declare that the research was conducted in the absence of any commercial or financial relationships that could be construed as a potential conflict of interest.
